# Association of weight and shape concern with weight change and weight-related behaviors in behavioral weight loss treatment

**DOI:** 10.1007/s10865-023-00451-5

**Published:** 2023-09-23

**Authors:** Stephanie P. Goldstein, KayLoni L. Olson, J. Graham Thomas

**Affiliations:** Department of Psychiatry and Human Behavior, The Miriam Hospital/Weight Control and Diabetes Research Center, Warren Alpert Medical School of Brown University, 196 Richmond St, Providence, RI 02903 USA

**Keywords:** Weight and shape concern, Body image, Behavioral weight loss, Weight change, Weight-related behaviors, Adherence

## Abstract

Weight and shape concern (WSC) is a facet of negative body image that is common among individuals with overweight/obesity seeking behavioral weight loss treatment (BWL), but remains understudied. This secondary analysis evaluates associations between WSC, weight change, and weight-related behaviors among individuals in a 24-week BWL. Adults (n = 32) with body mass index 25–50 kg/m^2^ completed a baseline WSC questionnaire, measured weight at 12 and 24 weeks, measured physical activity via accelerometer, and completed 24-hour dietary recalls. Adherence to self-monitoring dietary intake and weight were assessed. A series of linear mixed models were used to evaluate associations between baseline WSC and weight change, as well as weight-related behaviors. Results revealed no significant effect of WSC on weight change. There were significant WSC x time interactions, such that those rating WSC “very important” decreased self-weighing and the “low importance” group decreased their caloric intake during treatment. The “pretty important” group had greater minutes of activity than the “low importance” group. Findings indicated that WSC may impact weight-related behaviors that contribute to BWL success. This trial was pre-registered on ClinicalTrials.gov (NCT03739151).

## Introduction

Behavioral weight loss treatment (BWL) is recommended for individuals with a Body Mass Index (BMI) ≥ 25 kg/m^2^ to reduce risk for weight-related medical comorbidities (Curry et al., 2018). BWL targets sustainable changes to dietary intake and physical activity with evidence-based strategies (e.g., regular monitoring of diet, physical activity, and weight) to facilitate weight losses of 5–10% (Wadden et al., 2020). Body image concerns are a common source of motivation to pursue BWL among individuals of higher body weight (Poulimeneas et al., 2021). However, body image concerns may paradoxically interfere with healthy weight loss because they also reliably predict dysregulated eating and exercise behaviors (e.g., overeating, exercise avoidance; Stice & Van Ryzin, 2019).

Weight and shape concern (WSC), the degree to which sense of self-worth is influenced by body weight and/or shape, is a specific facet of body image concern relevant to individuals starting BWL (Wade et al., 2011). WSC is distinct from other body image constructs (i.e., body dissatisfaction) because it is more stable over time and places an individual at risk for feeling globally negative if their weight or shape is not consistent with their body ideals (Masheb et., 2006; Sharpe et al., 2018). Approximately 20% of weight management-seeking samples report high WSC (Dalle Grave et al., 2020; Olson et al., 2018) and high WSC when starting BWL has been associated with poorer weight loss (Olson et al., 2018; Wiedemann et al., 2021). Cognitive behavioral theories highlight the role of high WSC in disrupting weight-related behaviors, like eating and exercise goals or self-monitoring, and these disruptions vary across individuals (Grilo et al., 2019; Monteleone & Cascino, 2021). For example, some people with high WSC may engage in extreme caloric restriction while others may report overeating or cycling between restriction and overeating. Given the association of high WSC with reduced success in BWL, individuals of higher body weight may represent a group where the distress of high WSC makes it harder to engage in the behavioral changes necessary for weight loss including healthy caloric restriction, increased physical activity, and regular self-monitoring of these behaviors and body weight. However, this research question has not been formally tested.

The goals of this study are to: (1) replicate previous findings linking high WSC to lack of weight loss in BWL and (2) expand this research to investigate the association of baseline WSC with weight-related behaviors targeted in BWL. This is a secondary analysis of a 24-week BWL in which the primary aim was characterizing episodes of dietary non-adherence (Goldstein et al., 2021). It was hypothesized that those with high baseline WSC would exhibit lower weight losses at 12- and 24-weeks compared to those with low WSC and would demonstrate less frequent dietary self-monitoring, higher caloric intake, less frequent self-weighing, and fewer minutes of physical activity. Results, when appropriately replicated, may help identify WSC as a pre-treatment risk factor for reduced weight loss, which can ultimately guide the adaption of programs to improve BWL outcomes.

## Methods

### Participants

Self-selected participants from the community were recruited from October 2018 to September 2020. Inclusion criteria were: BMI of 25–50 kg/m^2^, aged 18–70 years, and diagnosis of ≥ 1 cardiovascular disease risk factor (e.g., Type 2 diabetes, hypertension). Exclusion criteria were: medical conditions contraindicating weight loss, pregnancy or breastfeeding within previous 6 months, enrollment in a weight loss program, weight loss of ≥5% in previous 6 months, taking weight loss medication, history of bariatric surgery, or diagnosed eating disorder, excluding Binge Eating Disorder. Participants were also asked to complete a 7-day run-in, in which the minimum criteria for starting treatment included tracking dietary intake (≥2 meals/day for 7 days) and gaining their physician’s confirmation of eligibility and permission to participate (see Goldstein et al., 2022 for details).

### Procedures

Participants provided written informed consent prior to data collection and treatment. The BWL consisted of 30-minute individual weekly sessions for the first 12 weeks and monthly sessions during the remaining 12 weeks. Treatment, informed by the Diabetes Prevention Program and Look AHEAD trials (Diabetes Prevention Program Research Group, 2002; Look AHEAD Research Group, 2003), included weight loss, calorie, and physical activity goals with behavioral and cognitive strategies to meet those goals. Self-monitoring of daily calorie intake, physical activity and weight was supported via MyFitnessPal. Participants were instructed to self-weigh no more than once per day (ideally in the morning) and this was used for treatment purposes (analyses used clinic weights). Sessions occurred in-person before transitioning to telephone due to the COVID-19 pandemic. Participants who completed the trial during the pandemic (n = 9) were asked to attend their final assessment in-person so they could be weighed by research staff. Procedures were approved by the Miriam Hospital Institutional Review Board (Board Reference #011617, Protocol ID: 1,089,492). The parent study was pre-registered on ClinicalTrials.gov (NCT03739151).

### Measures

*Weight and Shape Concern.* WSC was assessed at baseline with one item from a questionnaire of binge eating symptoms in Look AHEAD (Gorin et al., 2008; Look AHEAD Research Group, 2003): “During the past 6 months, has your weight or the shape of your body mattered to how you feel about yourself? Compare this to how you feel about other parts of your life—like how you get along with family and friends, and how you do at your job.” Response options were “not at all,” “somewhat,” “pretty,” or “very” important. This 1-item measure demonstrated validity in prior BWL samples (Olson et al., 2018). Due to few individuals in this sample rating WSC as “not important” (n = 2), the “not important” and “somewhat important” groups were combined into a “low importance” group for analysis. This practice is consistent with prior work (Olson et al., 2018).

*Weight and Height.* Height (mm) was measured at baseline with a wall-mounted stadiometer at the research center. Weight (kg) was measured to the nearest 0.1 kg using a calibrated digital scale at the research center. Percent weight loss was calculated at 12- and 24-weeks. Participants who declined an in-person final assessment due to COVID-19 were permitted to self-weigh at home (n = 2).

*Weekly Self-weighing Frequency*. Frequency of self-weighing was measured using ecological momentary assessment (EMA; self-report surveys delivered directly to participants’ smartphones). Using LifeData, a HIPAA-compliant smartphone application developed specifically to execute EMA protocols, participants completed 7 days of EMA every other week starting on week 1 (described in Goldstein et al., 2021). At the end-of-day survey (delivered randomly between 7-9pm), participants were asked “Did you weigh yourself today?”. Weekly frequency was calculated as total days a participant endorsed weighing themselves each week.

*Weekly Dietary Self-monitoring Frequency.* Participants were asked to monitor their diet daily using MyFitnessPal during the first 12 weeks of BWL (during weekly sessions), and were allowed to set their own goals for dietary self-monitoring in the final 12 weeks of BWL (during monthly sessions). MyFitnessPal entries were reviewed by clinicians weekly. Weekly frequency of dietary self-monitoring was calculated as total days a participant recorded a minimum intake of 200 calories during the initial 12 weeks of BWL (Turner-McGrievy et al., 2019). Dietary self-monitoring frequency during the latter 12 weeks of BWL was not included for analysis because the self-monitoring goal was not standardized across participants.

*Daily Caloric Intake.* Caloric intake was measured via 24-hour dietary recalls, a valid and reliable gold-standard in dietary assessment (Jonnalagadda et al., 2000). Trained research staff conducted phone-based recalls using the University of Minnesota Nutrition Data System for Research (NDSR) software (version 2018; Nutrition Coordinating Center, Minneapolis, MN, USA). During assessment weeks (1, 5, 11, 17, and 23), recalls were conducted on three random, non-consecutive days (2 weekdays, 1 weekend day). NDSR calculated daily caloric intake; usual daily intake was calculated by weighted average of daily intake estimates by weekend vs. weekday.

*Average Daily Moderate to Vigorous Physical Activity (MVPA) Minutes.* Physical activity was assessed via the ActiGraph GT9X Link (ActiGraph, LLC, Pensacola, FL, USA), featuring a triaxial accelerometer and validated data filtering algorithm, worn on the dominant wrist for 24 weeks. Weekly valid wear time was defined as ≥ 4 days with ≥ 8 h of wear per day, and nonwear periods (i.e., ≥ 90 min without movement) were removed (Schumacher et al., 2020). Previously established vector magnitude counts per minute thresholds for wrist-based ActiGraphs were used to estimate minutes of MVPA (≥ 7500 counts per minute; Kamada et al., 2016). Average daily minutes spent in MVPA per week was calculated.

### Statistical approach

Data were checked for outliers, normality, and heterogeneity, then analyzed with R studio version 4.0.2 (R Team, 2015). Associations between WSC level and percent weight loss (Aim 1) and weight-related behaviors (Aim 2) were evaluated with linear mixed models (“nlme package”; Pinheiro et al., 2013) with an autoregressive (AR(1)) covariance structure. Participants who discontinued BWL were not followed, per protocol (Goldstein et al., 2021); missing weight data was imputed using baseline data carried forward and all other missing data were handled via maximum likelihood estimation. Models included a random effect of subject, fixed effects of time and baseline WSC level (reference group: ‘low importance’ WSC rating), and WSC x time interaction term. Additional fixed covariates included: age, sex, and baseline BMI (percent weight loss model only); weekly percentage of EMA surveys completed (self-weighing model only); usual caloric intake (caloric intake model only); average daily ActiGraph wear-time (MVPA model only); and whether participants completed the trial during the COVID-19 pandemic (included when the effect of COVID-19 on the outcome was statistically significant). Statistically significant WSC x time interactions were graphed and probed using simple main effects contrasts (“emmeans” package; Lenth et al., 2018). Akaike information criterion (AIC) and conditional R^2^ demonstrate quality of model fit and the variance explained by each model, respectively. An a priori power analysis, described in Goldstein et al., 2021, was completed for the primary outcome of this trial (dietary non-adherence).

## Results

### Participant characteristics

This analysis included n = 32 enrolled participants (68.8% female, 75% white, 54.20 years (SD:10.7), BMI 38.38 kg/m^2^ (SD: 4.89)). Eighty individuals were screened for eligibility and 40 eligible individuals consented to participate. Eight participants did not successfully complete the 7-day run-in, leaving 32 enrolled participants. Of these 32, 15.6% (n = 5) dropped out of the study (see CONSORT in Goldstein et al., 2022). Baseline WSC levels were rated as ‘low importance’ (18.75%; *n* = 6), ‘pretty important’ (37.5%; *n* = 12), and ‘very important’ (43.75%; *n* = 14). See Tables 1 and 2 for participant characteristics and average weight loss, respectively.

**Table 1:** Participant Demographic Information by Baseline Weight and Shape Concern (WSC)

	Total Sample (n = 32)◊	Weight/Shape Low Importance (n = 6)	Weight/Shape Pretty Important (n = 12)	Weight/Shape Very Important (n = 14)		*p*-value*
Female, % (*n*)	68.8% (22)	83.3% (5)	66.7% (8)	64.3% (9)		0.69
Age, *M* (*SD*)	54.50 (10.70)	55.50 (9.27)	52.75 (12.02)	55.57 (10.54)		0.78
Baseline BMI, *M* (*SD*)	38.37 (4.89)	36.09 (3.37)	38.73 (6.04)	39.04 (4.35)		0.46
Race/Ethnicity						
White, % (*n*)	75.0% (24)	50.0% (3)	75.0% (9)	85.7% (12)		0.14
Black or African American, % (*n*)	9.4% (3)	33.3% (2)	0% (0)	7.1% (1)		
‘Other’, % (*n*)	15.6% (5)	16.7% (1)	25.0% (3)	7.1% (1)		
Hispanic or Latino, % (*n*)	18.8% (6)	16.7% (1)	33.3% (4)	7.1% (1)		0.23
Education						0.67
Less than bachelor’s degree, % (*n*)	28.13% (9)	33.3% (2)	41.7% (5)	14.3% (2)		
Bachelor’s degree or higher, % (*n*)	71.87% (23)	66.7% (4)	58.3% (7)	85.7% (12)		
Employment						0.54
Working full-time, % (*n*)	50.0% (16)	16.7% (1)	58.3% (7)	57.1% (8)		
Working part-time, % (*n*)	43.75% (14)	66.7% (4)	41.7% (5)	35.7% (5)		
Annual Income						0.56
<$50,000 per year, % (*n*)	31.25% (10)	66.7% (4)	16.7% (2)	28.6% (4)		
$50,000-$100,000 per year, % (*n*)	18.75% (6)	0% (0)	16.7% (2)	28.6% (4)		
>$100,000 per year, % (*n*)	43.75% (14)	33.3% (2)	58.3% (7)	35.7% (5)		
Prefer not to answer, % (*n*)	6.25% (2)	0% (0)	8.3% (1)	7.1% (1)		

*Significance testing of demographic variables across WSC level using chi-square test of independence (categorical variables) and ANOVA (continuous variables)

◊ANOVA and chi-square tests of independence evaluated differences between participants who completed the study during the COVID-19 pandemic (*n =* 9) vs. pre-COVID (*n* = 23). Participants completing the study during COVID-19 were more likely to be male (chi-square = 7.31, *p* = .006), likely due to directed attempts to diversify the sample towards the end of the study. There were no statistically significant differences by COVID-19 status in the remaining demographic variables.

**Table 2:** Weight loss and weight-related behaviors as a function of baseline Weight and Shape Concern (WSC)

			Statistical Test		
	12-week Average, M(SD)	24-week Average, M(SD)	WSC^a^	WSC x Time^a^	p-value of COVID-19 impact^d^
Percent Weight Loss^b^ _(positive value= weight loss)_	5.2 (3.8)	6.5 (6.1)	Pretty Important: B = 0.01, SE = 0.03, p = .77 Very Important: B = 0.002, SE = 0.03, p = .95	Pretty Important: B=-0.01, SE = 0.02, p = .75 Very Important: B=-0.01, SE = 0.02, p = .46	0.67
Weekly self-weighing frequency^c^	3.57 (2.56)	2.89 (2.38)	Pretty Important: B = 2.49, SE = 0.89, p = .008** Very Important: B = 3.56, SE = 0.87, p < .001***	Pretty Important: B=-0.05, SE = 0.04, p = .15 Very Important: B=-0.09, SE = 0.04, p = .01*	0.43
Weekly dietary self-monitoring frequency	5.73(2.25)		Pretty Important: B=-0.62, SE = 0.70, p = .38 Very Important: B=-0.13, SE = 0.69, p = .85	Pretty Important: B = 0.11, SE = 0.08, p = .17 Very Important: B = 0.12, SE = 0.08, p = .12	0.001**
Daily caloric intake^c^	1587.45 (378.54)	1387.27 (386.59)	Pretty Important: B=-213.49, SE = 104.20, p = .050* Very Important: B=-161.82, SE = 104.17, p = .13	Pretty Important: B = 28.43, SE = 10.14, p = .007** Very Important: B = 19.99, SE = 10.07, p = .051	0.19
Daily average MVPA minutes^c^	54.55 (31.47)	63.18 (35.33)	Pretty Important: B = 36.29, SE = 13.40, p = .01* Very Important: B = 15.43, SE = 13.09, p = .25	Pretty Important: B=-0.14, SE = 0.27, p = .59 Very Important: B=-0.21, SE = 0.28, p = .45	0.06

**Notes**: ^a^Reference category is “Low Importance”; ^b^missing data imputed as 0% weight loss (baseline weight carried forward); ^c^missing data handled using maximum likelihood estimation; ^d^indicator variable was created to distinguish participants who completed the trial during the COVID-19 pandemic (*n* = 9), and linear mixed models controlling for time and random effects of participant evaluated the effect of COVID-19 on weight change and weight-related behaviors. If the effect of COVID-19 was statistically significant, this indicator variable was included as a covariate in the model presented here; *p ≤ .05, **p < .01, ***p < .001

Percent weight loss observations: *n = 64;* represents ITT sample with 2 observations per participant; Self-weighing observations: *n* = 283; *M*(*SD*)_participant_ = 8.90(3.54); Dietary self-monitoring observations: *n* = 384; *M*(*SD*)_participant_ = 12.00(0.00); Caloric intake observations: *n* = 98; *M*(*SD*)_participant_ = 3.16(1.04); MVPA observations: *n* = 450; *M*(*SD*)_participant_ = 14.06(6.32)

### WSC and weight loss (Aim 1)

There was no statistically significant simple effect of WSC on percent weight loss, and no statistically significant time x WSC interaction (AIC=-212.84, conditional R^2^ = 0.78, Table 2).

### WSC and weight-related behaviors (Aim 2)

**Fig. 1:**
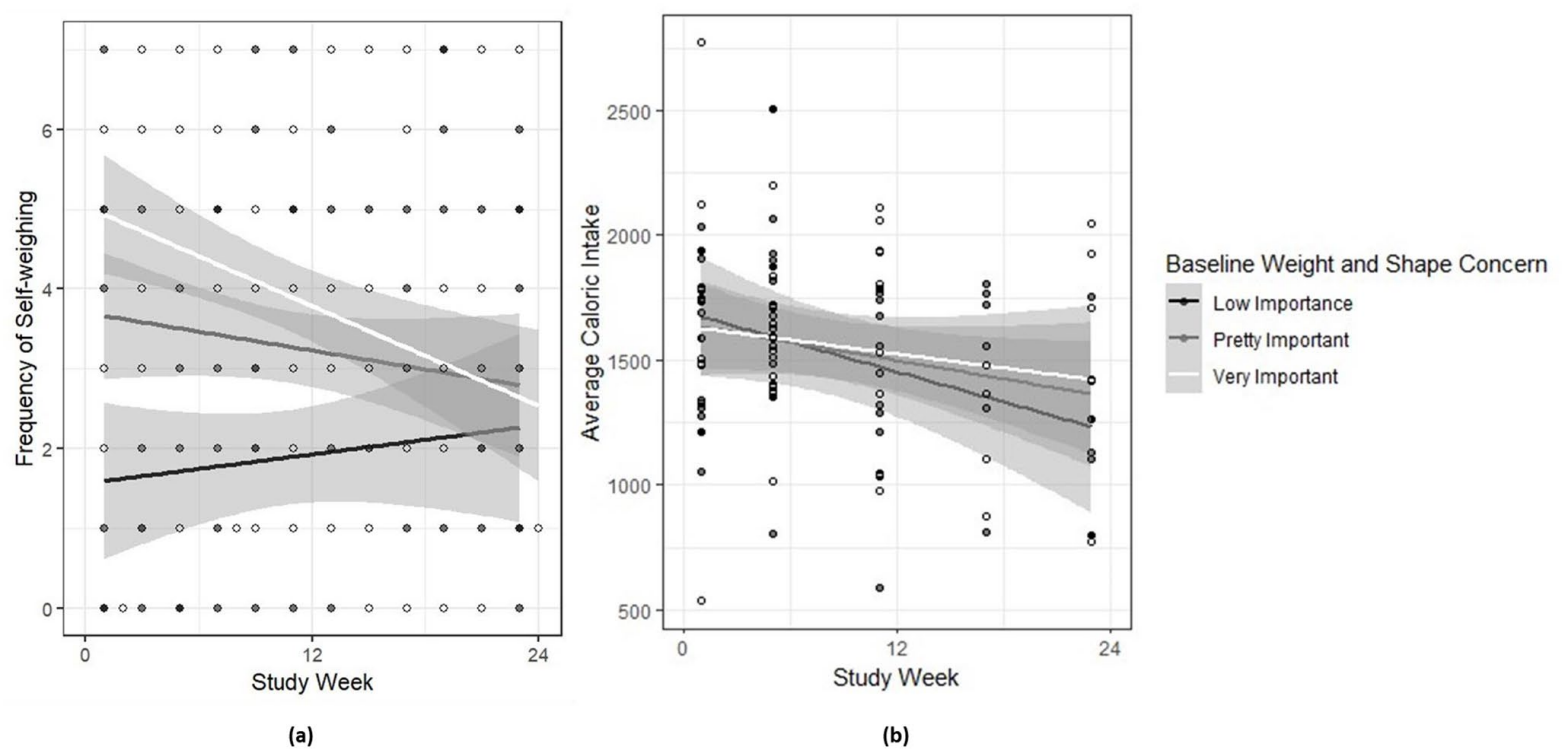
Illustration of frequency of self-weighning (panel a) and average caloric intake (panel b) across baseline levels of weight and shape concern (WSC)

Average self-weighing frequency, dietary self-monitoring frequency, daily caloric intake, and MVPA minutes are shown as a function of WSC in Table 2. There was a statistically significant WSC x time interaction on self-weighing (AIC = 1135.68, conditional R^2^ = 0.61). Post-hoc comparisons revealed that, at the outset of BWL, self-weighing frequency was greater among individuals rating WSC as “very important” (*B*=-3.46, *SE* = 0.85, *p* = .0003) and “pretty important” (*B*=-2.43, *SE* = 0.87, *p* = .009) compared to “low importance.” Additionally, there was a statistically significant decrease in self-weighing frequency over time when WSC was rated “very important” (*B* = 1.72, *SE* = 0.55, *p* = .002). Figure 1 (panel a) demonstrates linear trajectories of self-weighing frequency over time by baseline WSC. There were no statistically significant simple effects of WSC or WSC x time interaction effects on weekly dietary self-monitoring (AIC = 1636.01, conditional R^2^ = 0.29). There was a statistically significant WSC x time interaction on average daily caloric intake (AIC = 1377.66, conditional R^2^ = 0.54). Post-hoc comparisons revealed that, at the end of BWL, average daily caloric intake was greater among individuals rating WSC as “very important” (*B*=-298.17, *SE* = 150.10, *p* = .056) and “pretty important” (*B*=-440.44, *SE* = 152.7, *p* = .001) compared to “low importance.” Additionally, there was a statistically significant decrease in average daily caloric intake when WSC was rated as “low importance” (*B* = 627.94 *SE* = 188.20, *p* = .001). Figure 1 (panel b) demonstrates linear trajectories of average daily caloric intake over time by baseline WSC. There was a statistically significant simple effect of WSC, but no WSC x time interaction, on average daily MVPA minutes per week (AIC = 3712.37, conditional R^2^ = 0.85), with individuals who rated WSC as “pretty important” having more minutes of MVPA compared to the reference group (low importance). In sum, results evidenced statistically significant effects of WSC on many key behaviors that are related to weight (i.e., self-weighing frequency, daily caloric intake, and MVPA), but not on weight change, in this sample.

## Discussion

This is one of few studies to examine associations between WSC, weight, and weight-related behaviors during BWL. WSC was not associated with weight loss, which is inconsistent with previous research showing WSC to be associated with poorer weight loss (Olson et al., 2018; Wiedemann et al., 2021). However, WSC was associated with weight-related behaviors and this finding is consistent with prior work (Austin et al., 2017; Leung et al., 2017). While WSC was not associated with weight change in this study, the effect size was relatively large and there were significant associations between WSC and behaviors *related* to weight (e.g., caloric intake). It is possible that a statistically significant association between WSC and weight would be observed in a larger sample and over a longer BWL in which sustained weight-related behavior change would have a larger impact on weight. Overall, results provide promising early evidence of the effects of WSC on critical target behaviors during BWL.

In this study, individuals with the highest WSC self-weighed frequently (nearly daily, on average) at BWL outset, which decreased over time to be commensurate with the low importance group (3x per week). Negative body image has been associated with surveillance or body checking behaviors, such as self-weighing (Walker & Murray, 2012), which may be reflected in the high engagement with this behavior at the start of BWL for those with high WSC. However, there are many potential explanations for the patterns of self-weighing in this group that should be investigated in future studies including: natural regression to the mean, individuals mindfully switching to a more manageable self-weighing goal as they reach the maintenance phases of treatment, disengagement in treatment over time, or avoidance of weight-related information (Schumacher et al., 2021).

While there was no statistically significant effect of WSC on frequency of dietary self-monitoring, there was a significant WSC x time interaction on average daily caloric intake such that individuals rating WSC as “low importance” evidenced a decrease in caloric intake during BWL. Individuals rating WSC as “pretty” or “very” important reported higher caloric intake at the end of BWL, but these groups consumed an average daily caloric intake that is aligned with the treatment goals (1500–1800 kcals). This pattern may reflect increased engagement with BWL among those lower in WSC, or conversely, may reflect disengagement resulting in underestimation of intake. Given the limitations of self-reported intake, these results should be interpreted cautiously and underscore the importance of additional research. Future studies may consider examining state levels of WSC and intake, which could explain more variability and provide causal insights into daily eating behaviors (Panza et al., 2021).

Lastly, moderate WSC (“pretty important”) was associated with more physical activity minutes and this relationship did not change as a function of time in treatment. There is extensive evidence that body image is associated with physical activity engagement, likely in a bi-directional manner (Sabiston et al., 2019). However much of this work is conducted outside of the context of BWL. The current finding suggests that WSC at the start of treatment may be useful for identifying groups that require additional support for increasing physical activity.

Strengths of this secondary analysis include intensive longitudinal design and rigorous assessment procedures. There were several limitations including: COVID-related disruptions to data collection; bias of self-reported self-weighing frequency and caloric intake; drop-outs were not followed per study protocol, and thus results represent individuals who remain in BWL; small cell size of each WSC level; and a sample that was majority White female. Because the analysis plan did not formally account for multiple statistical tests (five models were evaluated), there is potential for Type 1 error; however, all findings were significant at *p* < .01 and thus would have remained statistically significant when applying a Bonferroni correction to control the family-wise error rate. There is also risk for Type 2 error, especially with regards to the effect of WSC on weight change, given the small sample size and fewer measured observations of the outcome (i.e.,12-week and 24-week weight change measures were used in contrast to the more frequent measurements of weight-related behaviors during treatment).

In sum, lower WSC at the start of BWL was associated with a more positive engagement profile as treatment progressed. The results of this study are largely hypothesis-generating, providing a signal that relationships between WSC, weight, and weight-related behaviors warrant further study. Replication with sufficiently powered and rigorously designed experiments is a necessary next step to clarify the role of WSC in the weight loss process. Given the potent motivator that negative body image is to pursue BWL, it is important to develop a nuanced understanding of how it influences behavior within these evidence-based treatments. Such research could inform the development of treatments that are tailored to baseline WSC level to maximize health benefits of weight loss.

## Data Availability

The corresponding author (SPG) is willing to provide all original, de-identified data for review upon written request.
